# Hospitalized pediatric drowning in Zhejiang, China (2010–2021): an epidemiological analysis

**DOI:** 10.1186/s12887-026-07320-5

**Published:** 2026-07-15

**Authors:** Shuzhen Zhu, Lijun Liu, Fengyun Chen, Xiuping Chen, Yanfen Lin, Caidi Zhang, Juya Ding, Sheng Ye, Kewen Jiang

**Affiliations:** 1 Emergency Department, Children’s Hospital Zhejiang University School of Medicine/National Clinical Research Center for Children and adolescents’ Health and Diseases, No. 3333 Binsheng Road, Hangzhou, 310053 China; 2 Department of Nursing, Children’s Hospital Zhejiang University School of Medicine/National Clinical Research Center for Children and adolescents’ Health and Diseases, No. 3333 Binsheng Road, Hangzhou, 310053 China; 3 Department of Child Psychology, Children’s Hospital Zhejiang University School of Medicine/National Clinical Research Center for Children and adolescents’ Health and Diseases, No. 3333 Binsheng Road, Hangzhou, 310053 China

**Keywords:** Children, Drowning, Drowning syndrome, Epidemiology, Prognosis

## Abstract

**Background:**

Despite advancements in public health awareness in China, pediatric drowning remains a significant concern with no marked decline in incidence. This study is to explore the changes in the epidemiological characteristics of children drowning.

**Methods:**

We retrospectively analyzed the clinical data of 296 drowning children who received treatment from January 2010 to December 2021.

**Results:**

We found that 1–3-year-old male children have the highest incidence of drowning in the afternoon of summer, especially in natural water in rural areas, which are more dangerous than swimming pools. The pre hospital cardiopulmonary resuscitation (CPR) time and drowning time are significantly associated with the outcome of the patient. In children without clear drowning time, our study found that clinical indicators such as Glasgow Coma Scale (GCS) score, blood lactate level, blood pH value, and blood glucose have a certain association with the outcomes of the children. The occurrence of child drowning incidents did not show significant changes due to the COVID-19 pandemic or the national control measures.

**Conclusions:**

Pediatric drowning persists as a critical public health issue, with rural areas posing the greatest risk. Early rescue and high-quality basic life support are key to improving outcomes. Targeted prevention strategies should prioritize rural water safety and caregiver education, while clinical indicators may aid in assessment of outcomes when drowning history is unavailable.

## Introduction

 Drowning, which involves respiratory impairment due to submersion or immersion in liquid, can cause significant damage to vital organs such as the brain, lungs, heart, and kidneys [1]. Globally, approximately 360,000 people die from drowning each year, with children accounting for about 45% of these fatalities. Drowning is recognized as one of the most preventable yet frequently overlooked public health issues and is the second leading cause of accidental deaths among children aged 0–14 worldwide [2]. In China, the drowning mortality rate for children is 8.77 per 100,000, with children aged 0–14 making up 56.04% of all drowning-related deaths [3]. Additionally, nonfatal drowning incidents are common among children, with about 90% of drowning cases involving children being nonfatal but requiring emergency care. More than 40% of these children need long-term hospitalization or further specialized treatment [4, 5]. Many of these nonfatal drowning survivors may suffer from severe neurological sequelae, including movement disorders, perceptual impairments, or cognitive deficits.

Drowning in children is influenced by a variety of factors [2, 6, 7]. Children aged 4–12 are particularly high-risk due to their limited cognitive ability to recognize danger [8]. Gender also plays a role, as males tend to be more active and engage in riskier behaviors compared to females. Seasonal patterns are evident, with a higher incidence during summer when outdoor water-related activities increase. Additionally, inadequate supervision in rural areas contributes to the risk. As public health awareness in China continues to improve, the epidemiological characteristics of child drowning may have shifted. However, there is currently a lack of large-scale data analysis to confirm these changes. To address this gap, this study retrospectively analyzed the clinical data of 296 drowning children admitted to the Children’s Hospital affiliated with Zhejiang University School of Medicine from January 2010 to December 2021. The aim is to understand the epidemiological trends of child drowning in the local area and provide insights to guide effective interventions, ultimately improving outcomes for these children.

## Methods

### Study design and participants

A retrospective study was conducted in the Emergency Department of the Children’s Hospital of Zhejiang University School of Medicine (Hangzhou, Zhejiang Province, China) during March 2023. The hospital serves pediatric patients from Zhejiang Province and neighboring regions. This study included pediatric drowning cases admitted to our hospital between January 2010 and December 2021. This was a retrospective, non-interventional study. As such, it was exempt from clinical trial registration requirements. The hospital’s ethics committee approved this study (Approval No. 2023-IRB-0255-P-01). Inclusion criteria comprised children aged 0–18 years with a confirmed history of drowning, while cases with incomplete medical records were excluded (32 cases). Patient data were extracted from the electronic medical record system using “drowning” or “drowning syndrome” as search terms. Collected variables included demographic characteristics, seasonal occurrence, drowning location, swimming proficiency, medical risk factors, drowning time, on-site resuscitation efforts, emergency evaluation findings, laboratory test results, and hospital management protocols. Data collection was performed by one researcher and independently verified by another. Any discrepancies were resolved through team consensus to ensure data accuracy. Patient outcomes were assessed using the Pediatric Cerebral Performance Category Scale (PCPC), which categorizes neurological status into six levels ranging from 1 (normal) to 6 (brain death), with higher scores indicating more severe cerebral impairment [9, 10].

### Statistical analysis

EmpointStats 5.0 and SPSS 26.0 were utilized for statistical analysis. Continuous data conforming to a normal distribution (as determined by the Kolmogorov-Smirnov test) were expressed as means ± standard deviation (SD), while non-normally distributed data were expressed as means ± standard error of means (SEM). Categorical data were presented as frequencies (n) and percentages (%). Factors associated with the outcome of drowning children were analyzed using general linear regression, with the PCPC score as the dependent variable. Smooth curve fitting was employed to identify potential variables associated with outcome, including drowning time, prehospital CPR duration, in-hospital GCS score, blood lactate level, blood pH value, and blood glucose level. Comparisons of each indicator across different years were performed using the chi-square test or one-way ANOVA. A *P*-value < 0.05 was considered statistically significant, and a correlation coefficient |R| > 0.3 was deemed indicative of a linear relationship.

## Results

### Demographics and prehospital clinical characteristics

A total of 296 children were included in the study with an average of 24.67 ± 8.47 cases per year. The annual cases ranged from a minimum of 7 cases in 2010 to a maximum of 43 cases in 2019. Among 296 drowning children, 211 (71.3%) were males and 85 (28.7%) were females. More than half of the drowning incidents involved young children aged 1-3y (74.7%), and the incidence rate in rural areas (79.9%) was significantly higher than in urban areas. Drowning incidents predominantly occurred in the afternoon (40.2%) and during the summer months (34.1%.). The majority of cases were accidental (93.2%), with only 2 cases involving suicidal tendencies and 18 cases occurring during swimming activities. The most common locations for drowning were natural water bodies, particularly ponds (53.0%) and small rivers (26.7%). See Table 1.

**Table 1:** Demographics and clinical characteristics of drowning children (*n* = 296)

Characteristic	*N*(%)
Year	296(100)
2010	7(2.4)
2011	20(6.8)
2012	21(7.1)
2013	25(8.4)
2014	29(9.8)
2015	19(6.4)
2016	28(9.5)
2017	23(7.8)
2018	25(8.4)
2019	43(14.5)
2020	25(8.4)
2021	31(10.5)
Age	
Infant (0–12 m)	8(2.7)
Toddler (1-3y)	221(74.7)
Preschool (4-6y)	31(10.5)
School (7-12y)	35(11.8)
Adolescent (13-18y)	1(0.3)
Gender	
Male	211(71.3)
Female	85(28.7)
The only child	
Yes	97(32.8)
No	199(67.2)
Living area	
Village	236(79.7)
City	60(20.3)
Medical risk factors	
Yes	2(0.7)
No	294(99.3)
Swimming ability	
Pre-swimming age (<6y)	249(84.1)
Can swim	10(3.4)
Can not swim	37(12.5)
Drowning time	
7: 00–12༚00	84(28.4)
12: 01–17༚00	119(40.2)
17: 01-morrow 6༚59	93(31.4)
Drowning season	
Spring (3-5month)	77(26.0)
Summer (6-8month)	101(34.1)
Autumn (9-11month)	63(21.3)
Winter (12-2month)	55(18.6)
Drowning on school day	
Yes	195(65.9)
No	101(34.1)
Drowning way	
When swimming	18(6.1)
Accidental	276(93.2)
Suicide	2(0.7)
Drowning location	
Pond	157(53.0)
Small river	79(26.7)
Bathtub	29(9.8)
Swimming pool	17(5.7)
Other location	14(4.8)
Witnessed drowning	
Yes	114(38.5)
No	182(61.5)
Drowning event location to the emergency room transportation mode	
Ambulance	185(68.5)
Private car	111(37.5)
Prehospital CPR	
Non-essential	26(8.8)
Performed	251(84.8)
Not performed	19(6.4)
Prehospital CPR time[min, M(P_25_,P_75_)] ^b^	18.89(3.0, 30.0)
Drowning time (min, M ± SD)^c^	7.06 ± 6.17

^a^ Autism, arrhythmia and epilepsy are risk factors for drowning, ^b^ Missing *n* = 72, ^c^ Missing *n* = 73

**Fig. 1:**
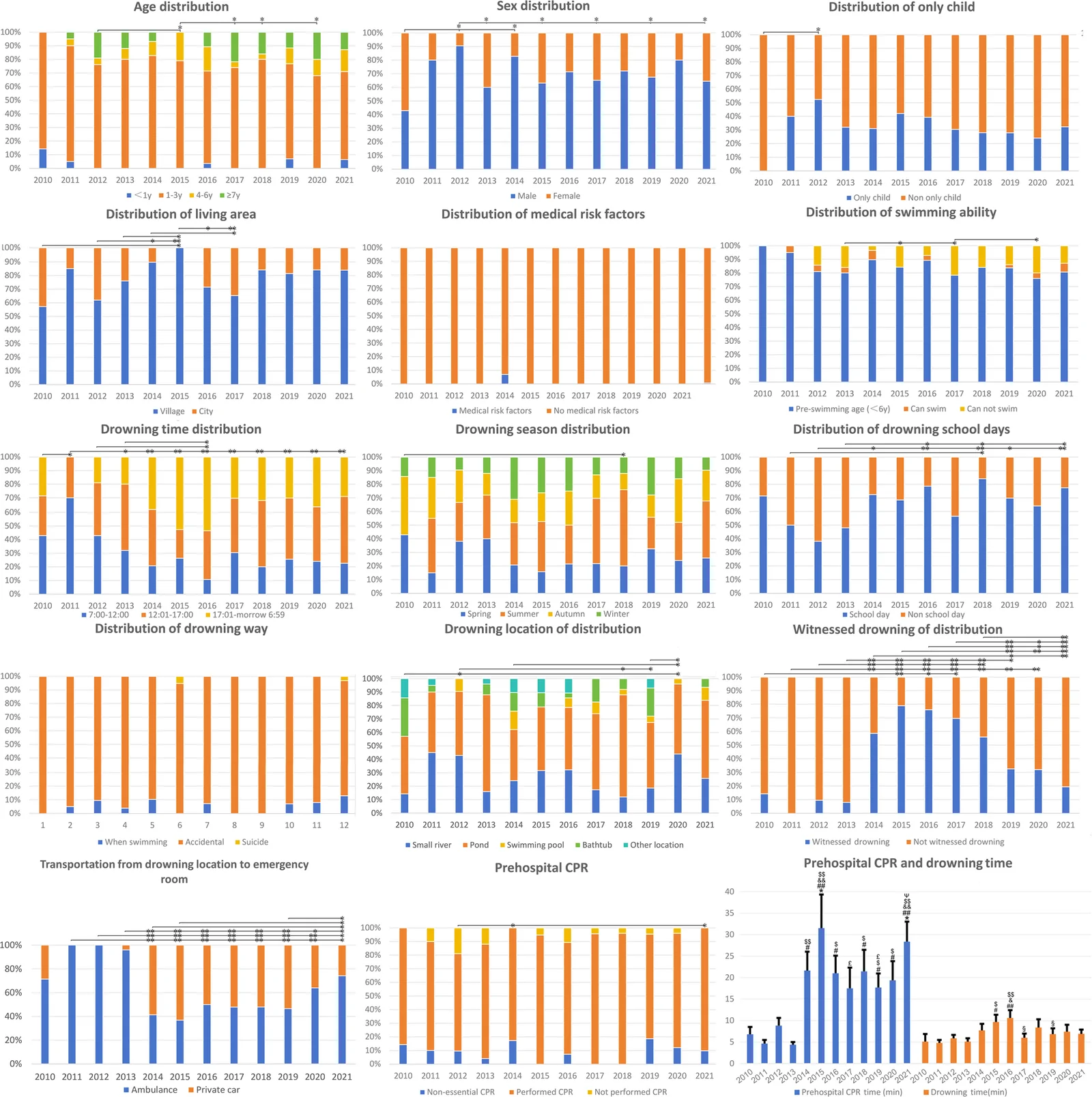
The dynamic trend of demographics and prehospital clinical characteristics in drowning children of different years. The comparisons of each indicator between years were conducted using chi square test or One-Way ANOVA analysis. ^*^p < 0.05, ^**^ *p* < 0.001; For prehospital CPR and drowning time, ^*^ *p* < 0.05 compared to 2010; ^#^*p* < 0.05, ^##^ *p* < 0.001 compared to 2011; ^&^ *p* < 0.05, ^&&^ *p* < 0.001 compared to 2012; ^$^ *p* < 0.05, ^$$^ *p* < 0.001 compared to 2013; ^£^ *p* < 0.05 compared to 2015; ^§^*p* < 0.05 compared to 2016; ^Ψ^*p* < 0.05 compared to 2019

The dynamic trend of children drowning over the years are showed in Fig. 1. Key observations are summarized below:

Living area: Village-based drownings consistently outnumbered city-based cases in all years. 2015 was unique, with all cases occurring in villages, differing significantly (*P* < 0.05) from 2010, 2012, 2013, 2016, and 2017. Additionally, 2014 showed significant differences compared to 2012 and 2017. Drowning Time: 2011: All incidents occurred between 7:00 AM and 5:00 PM, differing significantly (*P* < 0.05) from 2010, 2013, and 2021. 2016: Markedly fewer cases occurred between 7:00 AM and 12:00 PM compared to 2012 and 2013 (*P* < 0.05). Drowning Season: 2010 was unique for having no summer cases, differing significantly (*P* < 0.05) from 2018. School Days vs. Non-School Days: Most years reported more drownings on school days, except 2011, 2012, and 2013. Significant differences were observed: 2011 vs. 2018; 2012 vs. 2014, 2016, 2018, 2019, and 2021; 2013 vs. 2016, 2018, and 2021 (*P* < 0.05). Drowning Location: Ponds were the most common location annually, followed by small rivers and bathtubs. Significant differences included: 2010 (no bathtub cases) vs. 2012 and 2020; 2012 vs. 2018 and 2019; 2020 vs. 2014 and 2019 (*P* < 0.05). Prehospital CPR: CPR was administered in the majority of cases. 2012 differed significantly from 2014 to 2021, as no cases in 2012 lacked CPR. Prehospital CPR duration increased significantly after 2014.

### Data for in-hospital treatment

A total of 119 patients (40.2%) were recovered and discharged, 76 patients (25.7%) died, and 101 patients (34.1%) needed further rehabilitation. Among 238 children (80.4%) were admitted to the ICU, with an average duration ICU stay of 6.38 ± 5.68 days (see Table 2).

**Table 2:** Hospital treatment of drowning children

Characteristic	*N*(%)
Intra-hospital endotracheal intubation	
Yes	158(53.4)
No	138(16.6)
Intra-hospital continuing CPR	
Yes	141(47.6)
No	155(52.4)
Chest X-ray	
Normal	40(13.5)
Bilateral infiltrate	256(86.5)
PICU	
Yes	238(80.4)
No	58(19.6)
Discharge overview	
Recovery	119(40.2)
Rehabilitation	101(34.1)
Die	76(25.7)
PCPC(score)	
1	108(36.4)
2	29(9.8)
3	15(5.1)
4	27(9.1)
5	41(13.9)
6	76(25.7)
Hospital GCS score (score, M ± SD)	8.43 ± 5.20
Lactic acid [mmol/L, M(P_25_,P_75_)]	4.40(1.30, 5.28)
Blood sugar (mmol/L, M ± SD)	10.00 ± 6.70
PH value (M ± SD)	7.27 ± 0.15
PICU HOD (d, M ± SD) ^a^	6.38 ± 5.68

^a^ Missing *n* = 58

**Fig. 2:**
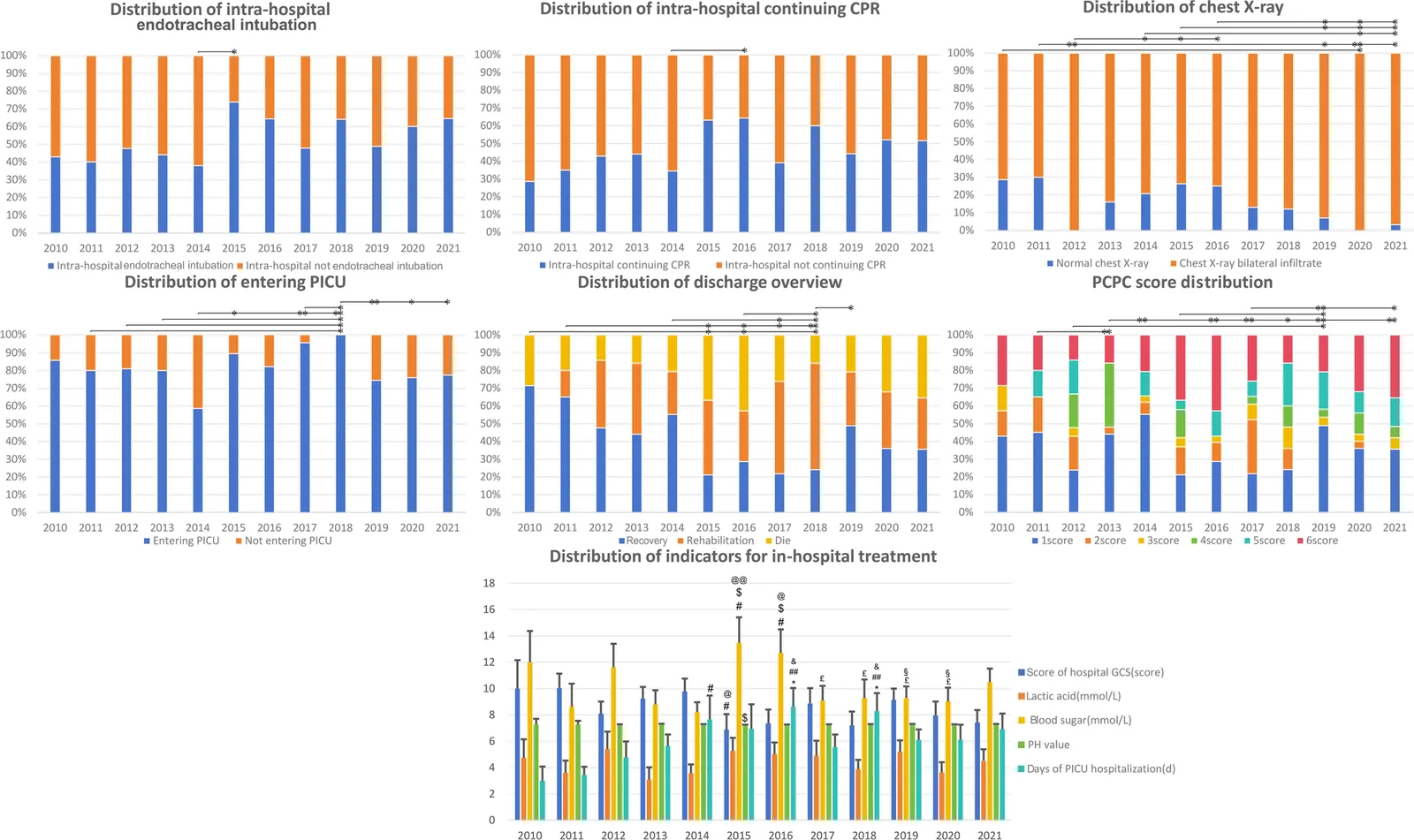
The dynamic trend of in-hospital clinical characteristics in different years. The comparisons of each indicator between years were conducted using chi square test or One-Way ANOVA analysis. ^*^ *p* < 0.05, ^**^ *p* < 0.001; For distribution of indicators for in-hospital treatment, ^*^ *p* < 0.05 compared to 2010; ^#^*p* < 0.05, ^##^ *p* < 0.001 compared to 2011; ^&^ *p* < 0.05 compared to 2012; ^$^ *p* < 0.05 compared to 2013; ^@^*p* < 0.05, ^@@^*p* < 0.001 compared to 2014; ^£^ *p* < 0.05 compared to 2015; ^§^*p* < 0.05 compared to 2016

The dynamic changes in in-hospital treatments across years are illustrated in Fig. 2. For intra-hospital endotracheal intubation, the proportion of patients requiring this intervention showed an upward trend in 2015, with a significant difference observed between 2014 and 2015. Correspondingly, the proportion of patients requiring intra-hospital continuing CPR increased after 2015, and 2014 differed significantly from 2016 (*P* < 0.05). In contrast, the proportion of patients undergoing chest X-ray examinations declined steadily from 2016 onward. The proportion of patients admitted to the Pediatric Intensive Care Unit (PICU) peaked in 2017 and 2018. Notably, while 2018 had the highest proportion of cases requiring rehabilitation, it did not exhibit the highest mortality rate or the lowest cure rate among the studied years.

### Factors affecting the prognosis of drowning in children

Using PCPC score as the dependent variable, the associations between various clinical parameters and outcomes are analyzed [11–17]. A general linear analysis was performed, with *P* value < 0.05 indicating statistical significance and | R |>0.3 indicating a linear correlation. The results demonstrated that the Glasgow Coma Scale (GCS) score and requirement of in-hospital CPR were strongly associated with drowning outcomes in this cohort (|R| ≥ 0.8). Prehospital CPR duration, drowning time, blood lactate levels, and blood glucose levels showed moderate associations with outcomes (|R| fluctuates between 0.5 and 0.8). Blood pH and prehospital CPR administration status exhibited weak associations with outcomes (|R| between 0.3 and 0.5), while other variables showed no significant linear association (|R| < 0.3). Detailed results are presented in Table 3.

**Table 3:** General linear regression analysis of prognostic factors affecting childhood drowning

Characteristic	*R*	95%CI (Lower)	95%CI (Upper)	*P* value	t value
Hospital GCS score	-0.93	-0.95	-0.92	0.000	-45.125
Intra-hospital continuing CPR	-0.88	-0.90	-0.85	0.000	-31.647
Prehospital CPR time (min)	0.72	0.65	0.78	0.000	15.489
Lactic acid (mmol/L)	0.56	0.48	0.64	0.000	11.719
Blood sugar (mmol/L)	0.54	0.45	0.61	0.000	10.950
Drowning time (min)	0.57	0.47	0.65	0.000	10.205
PH value	-0.49	-0.57	-0.40	0.000	-9.671
Prehospital CPR	0.37	0.27	0.47	0.000	6.858
Age (y)	0.04	-0.07	0.16	0.453	0.751
Gender	0.03	-0.08	0.14	0.601	0.523

### Smooth curve fitting for risk factors

**Fig. 3:**
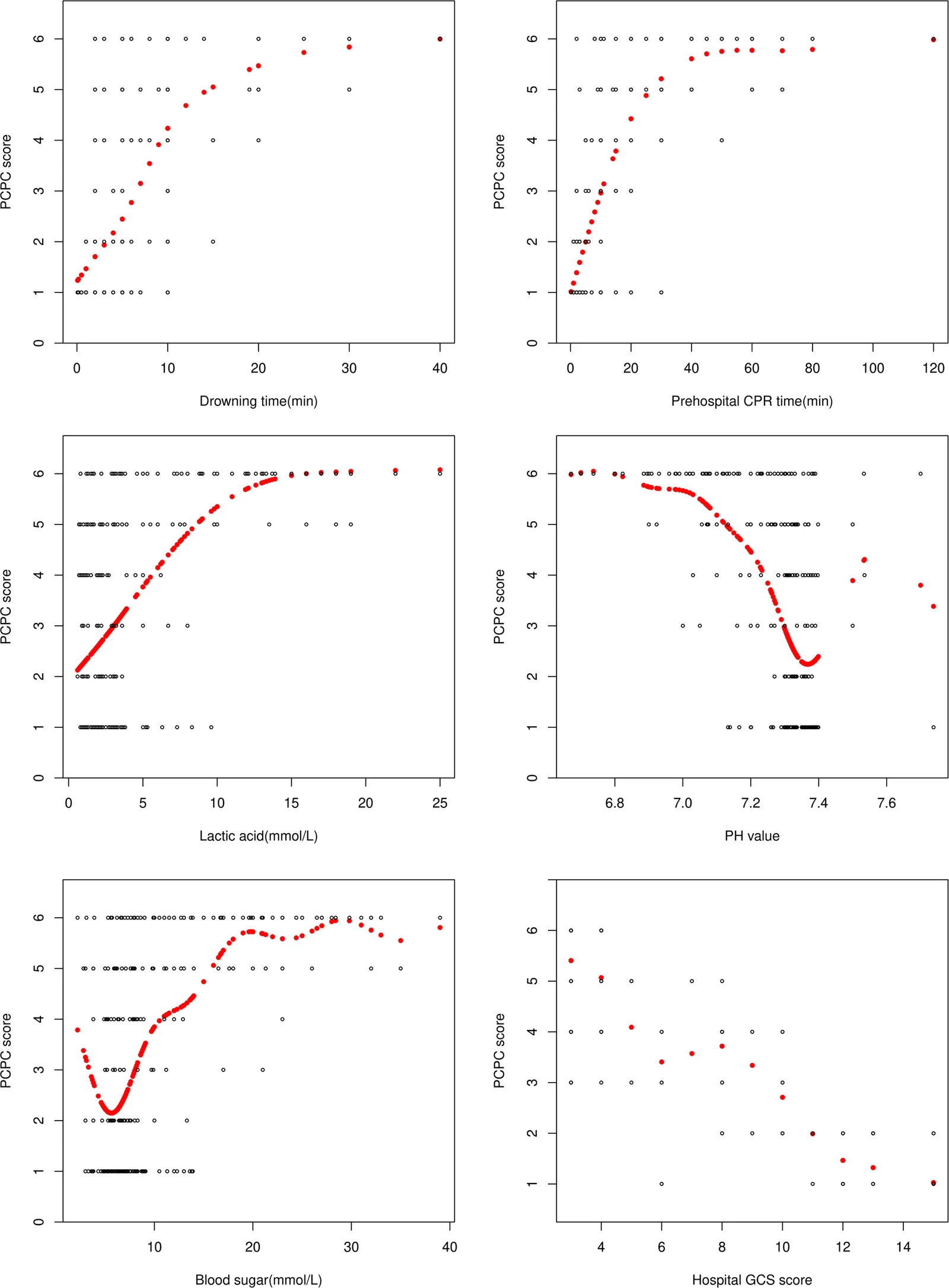
Smooth curve fitting analysis of variables associated with outcome in this cohort of drowning children. The red line represents the fitting curve, and the small black dots represent the distribution of the data

Smooth curve fitting was applied to continuous variables associated with outcome in this cohort (drowning time, pre-hospital CPR time, blood lactate level, GCS score, blood glucose, and blood pH). The red line illustrates the fitted curve, while the blue line indicates the 95% confidence interval for the association between each variable and the PCPC score of the drowning child. It should be noted that the PCPC is an ordinal categorical scale; treating it as a continuous variable for analysis may simplify the complexity of clinical distances. Therefore, the linear correlations and smooth curve fittings presented in this study should be interpreted as exploratory trends rather than precise quantitative predictive models. See Fig. 3.

## Discussion

Globally, drowning incidents occur most frequently in children aged 1–4 years, followed by those aged 5–9 years [18, 19]. Our study identified 296 drowning cases, with 74.7% (*n* = 221) involving toddlers (1–3 years), followed by preschoolers (4–6 years) at 10.5% (*n* = 31) and school-aged children (7–12 years) constituting 11.8% (*n* = 35) of cases. This peak vulnerability in early childhood likely stems from their developmental stage of intensive environmental exploration, coupled with immature cognitive capacity to assess risks, particularly when unsupervised. The observed male predominance (2.5:1 gender ratio) aligns with WHO’s 2014 drowning surveillance data [20], potentially attributable to males’ generally higher activity levels and more pronounced risk-taking behaviors compared to females [21]. These findings underscore the need for enhanced supervisory measures for male children within these critical developmental stages.

Chen Yichen et al. [22] demonstrated seasonal patterns in pediatric drowning incidence. Our analysis revealed summer as the peak occurrence period (34.1%), primarily during afternoon hours (40.2%), with spring (26.0%) and autumn (21.3%) following sequentially – a distribution pattern corroborated by existing epidemiological studies [18, 23]. These temporal trends correlate strongly with elevated summer temperatures and corresponding increases in children’s unsupervised aquatic activities during daylight hours. Notably, 80% of drowning incidents in our cohort occurred in natural waterways (small rivers/ponds), contrasting sharply with swimming pool-related cases (5.7%) – findings aligned with domestic research [24] but discordant with international studies reporting over 50% pool-related drowning events [11, 12]. This geographical discrepancy underscores the critical need for targeted public education campaigns and enhanced protective infrastructure around China’s natural water bodies.

Our analysis revealed significantly higher drowning incidence during school days (65.9%) versus holidays (34.1%), contrasting with Australian epidemiological findings on school-age aquatic mortality [25]. This discrepancy may stem from our cohort’s predominant rural distribution (79.7%) - environments with greater natural water exposure risks. During academic weekdays, working parents typically delegate childcare to grandparent caregivers whose compromised supervision capabilities (inadequate crisis recognition capacities, age-related cognitive limitations, and safety complacency) significantly elevate drowning risks [26]. Notably, 61.5% of incidents involved complete guardian absence, aligning with global surveillance data [11, 12, 27]. Existing evidence demonstrates that enhanced supervision protocols coupled with aquatic environmental modifications could prevent over 85% of such tragedies [22]. The school day risk elevation proves particularly acute in rural contexts, where unsupervised children face dual vulnerabilities: environmental exposure and delayed emergency response systems compared to urban areas [28]. These findings collectively highlight the urgent requirement for (1) community-based caregiver education programs addressing developmental supervision needs, (2) implementation of comprehensive caregiver certification systems, and (3) reorientation of drowning prevention strategies to prioritize rural infrastructure development and emergency medical network optimization.

Drowning typically results in fatal outcomes within 4–6 min, with pediatric survival rates critically dependent on two temporal factors: the interval between submersion and water removal, and the initiation timing of appropriate resuscitation measures [29]. The implementation of standardized pre-hospital emergency protocols demonstrates significant improvements in outcomes [30]. Our findings identify four four variables significantly associated with clinical outcomes (*P* < 0.05): (1) hospital-based CPR continuation requirement; (2) prehospital CPR duration (time-to-admission interval); (3) total drowning time; and (4) prehospital CPR administration status. The PCPC score shows associations with temporal patterns: PCPC scores tend to increase with drowning time ≤ 10 min, followed by a diminishing association between PCPC score and drowning time in the 10–20 min range. Beyond 20 min of drowning time, the PCPC score does not show a significant further increase, exhibiting a trend toward stabilization, suggesting that the stabilization of the neurologic score is linked to the higher score/worse outcome with the higher drowning times. While PCPC scores show a positive association with prehospital CPR duration, this association becomes nonlinear when CPR exceeds 30 min - a pattern consistent with existing literature [11–13]. This deviation may be related to China’s suboptimal public CPR training rates (38.3% awareness vs. 11.4% practical competency in national surveys), which may contribute to delayed high-quality resuscitation as untrained bystanders await professional responders. The observed clinical deterioration associated with prolonged drowning and extended CPR duration is consistent with two pathophysiological consequences: cumulative cerebral hypoxic-ischemic damage and secondary reperfusion injuries. These findings suggest two critical intervention points: (1) immediate high-quality basic life support implementation, and (2) systematic public education programs targeting early recognition and evidence-based CPR techniques to optimize neurological preservation.

Central nervous system injury is a critical factor associated with mortality and morbidity following drowning, with drowning time emerging as a parameter notably associated with outcome in this cohort [14]. While our study demonstrated a notable association between drowning time and outcome (|R|=0.57), this association proved weaker compared to the intra-hospital continuous CPR indicator (|R|=0.88). This discrepancy likely stems from the inherent limitations of drowning time data, which primarily relies on estimations from witnesses and consequently may be subject to inaccuracies or incompleteness. Therefore, our investigation incorporated pertinent clinical objective indicators post-admission. Our findings identified statistically significant correlations (*P* < 0.05) between poor outcomes and several clinical parameters: GCS score, blood lactate level, blood pH value, and blood glucose levels, consistent with previous studies [11, 12, 15, 16]. GCS scores showed a notable association with outcome in the context of brain injury. Our analysis revealed a significant inverse correlation between GCS and PCPC (Pediatric Cerebral Performance Category) scores (|R|=0.93). This finding further suggests the potential value of GCS in this cohort: as GCS scores decrease—particularly when falling below 8—the risk of adverse outcomes significantly increases, and patients are more likely to experience severe brain injury or death. Notably, patients with GCS < 8 and PCPC ≥ 3 exhibited poorer outcomes, underscoring severe coma (GCS < 8) as a critical clinical indicator associated with unfavorable outcomes.Blood lactate level, serving as a sensitive marker of peripheral tissue perfusion and cellular hypoxia, demonstrated distinct patterns of association with outcome : In the low lactate range (< 10mmol/L), PCPC scores increased with rising lactate levels (higher lactate was associated with more severe hypoxia and worse outcome). However, when lactate levels > 10mmol/L, PCPC scores plateaued and no longer rose with further increases in lactate, suggesting a threshold beyond which more complex metabolic derangements may emerge.The blood pH, as a core indicator of systemic acid-base balance, exhibits a significant negative correlation with outcome severity. The decline in pH is associated with hypoxia and is considered marker of metabolic acidosis. Our observations suggest that lower blood pH levels correspond to higher PCPC scores, consistent with more severe acidosis and a markedly increased risk of adverse outcomes. Regarding blood glucose levels, we identified distinct outcome-related patterns: When blood glucose reaches 5 mmol/L, the PCPC score was at its lowest, corresponding to the best outcome. Both hypoglycemia (< 5 mmol/L) and hyperglycemia (> 5 mmol/L) are associated with worsening outcomes, demonstrating a U-shaped risk pattern. Specifically, hypoglycemia typically is associated with severe energy depletion or metabolic derangement, whereas hyperglycemia (5–20 mmol/L) may indicate stress responses, systemic inflammation, or insulin resistance—factors that are associated with worsened hypoxia and increased brain injury. When blood glucose exceeds 20 mmol/L, the PCPC score plateaus, suggesting a possible saturation effect in glucose metabolism or the predominance of other mortality/disability mechanisms. These findings suggest that in cases with uncertain drowning times, outcomes can be evaluated with caution through these clinical indicators, which may offer a potentially useful reference for pediatric drowning victims.

Since the outbreak of the COVID-19 pandemic in 2020, China implemented a dynamic zero-COVID strategy until late 2022 to early 2023, after which the country gradually lifted restrictions and officially adjusted its management measures in January 2023. However, the occurrence of child drowning incidents did not show significant changes due to the COVID-19 pandemic or the national control measures. This also corroborates our findings. The reasons may be multifaceted: (1) Pronounced Seasonal Characteristics: Drowning incidents exhibit distinct seasonal patterns, primarily concentrated during the high-temperature summer months, which creates a temporal misalignment with pandemic control cycles. Adjustments to prevention and control measures were focused during winter (late 2022 to early 2023), showing limited relevance to the peak drowning season. (2) Limited Effectiveness of Routine Family Supervision: While home isolation may have increased the time parents spent with their children, the quality of supervision did not significantly improve with extended duration. Some parents still had to balance work responsibilities, and there was no fundamental shift in their prioritization of children’s safety education or vigilance around water bodies. (3) Persistent Gaps in Hazardous Water Area Management: Longstanding deficiencies in safety infrastructure, such as inadequate drowning prevention signage, broken barriers, and insufficient protective measures around open water bodies like ponds and rivers in rural areas, remain unaddressed despite the pandemic. These unresolved risks continue to accumulate. (4) Uneven Coverage of School-Based Drowning Prevention Education: Online teaching during the pandemic weakened the effectiveness of safety education, with rural areas further hindered by poor internet connectivity, resulting in incomplete dissemination of drowning prevention knowledge. Post-reopening, schools failed to promptly reinforce safety education curricula, leaving students’ safety awareness unaligned with the intensity of pandemic controls. Persistent water hazards, chronic gaps in supervision, and insufficient safety investments underscore the necessity for establishing a long-term mechanism for child drowning prevention. Short-term public health policies alone are unlikely to yield direct impact.

### Limitations

Firstly, this study was conducted at a specialized pediatric hospital (tertiary Grade A), where the majority of patients were referred drowning cases from primary hospitals. As a result, the findings may not be fully generalizable to the broader population. Future research should involve collaboration with multiple medical centers to conduct multicenter studies, ensuring more comprehensive and representative results.

Secondly, due to the retrospective nature of this study, there were limitations in the availability of sufficient patient medical history records, with some data being incomplete, particularly regarding drowning events. This restricts the ability to analyze potential risk factors influencing drowning incidence, such as parental education levels and safety measures near water areas.Due to the retrospective nature of this study, data on some key confounding variables, such as water temperature, were unavailable. Therefore, multivariable regression analysis could not be performed to adjust for these confounders. Consequently, healthcare professionals must recognize the critical importance of improving the completeness and accuracy of medical records. Enhanced documentation would enable a more precise description of the epidemiology of pediatric drowning and facilitate the development of targeted interventions. Additionally, our study lacked long-term follow-up for discharged patients with severe neurological injuries. Furthermore, there were incomplete records of diagnostic assessments such as head imaging, head ultrasound, electroencephalogram, and other related outcomes following drowning. These indicators are potentially correlated with outcome and could provide valuable insights into long-term neurological outcomes. Addressing these gaps in future studies would strengthen the understanding of factors associated with outcome and improve clinical management strategies for pediatric drowning cases.

## Conclusions

This 12-year retrospective single-center study of pediatric drowning in Zhejiang Province identified a predominant demographic of male toddlers (1–3 years; 74.7% of cases) with peak incidence in rural natural waters during summer afternoons. The study highlighted that the key to mitigating the harm caused by drowning lies in prevention, which necessitates collaborative efforts from families, schools, and society.

Our findings underscore that child drowning intervention strategies should prioritize rural areas, where the risk appears to be most pronounced. Notably, prehospital CPR duration and drowning time were significantly associated with patient outcomes, underscoring the critical importance of early detection and high-quality basic life support in improving outcomes. In cases where drowning time is unclear, clinical indicators such as GCS score (|R|=0.94), blood lactate level, blood pH value, and blood glucose levels exhibit associations with outcomes, providing potentially useful tools for assessing outcomes in pediatric drowning victims.

The occurrence of child drowning incidents did not show significant changes due to the COVID-19 pandemic or the national control measures, revealing persistent systemic gaps in prevention. These insights emphasize the need for continued research, improved public awareness, and targeted interventions to reduce drowning-related morbidity and mortality.

Recommendations:

1. Targeted Environmental Modifications: Urgent infrastructure improvements (protective barriers, warning systems) are needed for high-risk rural water bodies.
2. Comprehensive Public CPR Training: Nationwide first-aid training initiatives are essential to enhance the quality of bystander CPR.
3. Public Health Integration: Drowning prevention should be integrated into sustainable public health strategies.

### Study limitations

The single-center design and retrospective methodology limit the generalizability of the findings, suggesting a need for multicenter validation studies.

## Data Availability

The clinical data supporting the findings of this study are available from the corresponding author at [zsz727@zju.edu.cn] upon reasonable request.

## Abbreviations

COVID-19: Coronavirus disease 2019
PCPC: Pediatric Cerebral Performance Category
GCS: Glasgow Coma Scale
